# A Comparison of Cooking Conditions of *Rhizoclonium* Pulp as a Substitute for Wood Pulp

**DOI:** 10.3390/polym14194162

**Published:** 2022-10-04

**Authors:** Pai-An Hwang, Song-Ling Wong, Yu-Ching Liu

**Affiliations:** 1 Department of Bioscience and Biotechnology, National Taiwan Ocean University, Keelung 202031, Taiwan; 2 Center of Excellence for the Oceans, National Taiwan Ocean University, Keelung 202301, Taiwan; 3 Department of Raw Materials and Fibers, Taiwan Textile Research Institute, New Taipei City 23674, Taiwan

**Keywords:** *Rhizoclonium*, sodium hydroxide, sodium sulfite, hydrogen peroxide, pulp, substitutes, mechanical property

## Abstract

The green macroalga *Rhizoclonium* was cooked with 5%, 10%, and 20% sodium hydroxide (NaOH) for 4 h (5-N, 10-N, and 20-N groups, respectively); with 5%, 10%, and 20% sodium sulfite (Na_2_SO_3_) for 4 h (5-NS, 10-NS, and 20-NS groups, respectively); and with 5%, 10%, and 20% NaOH for 2 h and 1% hydrogen peroxide (H_2_O_2_) for 2 h (5-NH, 10-NH, and 20-NH groups, respectively). The 5-NH handsheet showed the best mechanical properties; however, the 10-NH pulp was easier to separate than 5-NH during handsheet making, and 10-NH was more suitable for the industrial process. Thus, the 10-NH group showed the optimal production conditions with an optimal length/width ratio, crystallinity index (CI%), three-dimensional (3D) configuration, and mechanical strength. Substituting 20% 10-NH *Rhizoclonium* pulp with wood pulp had no significant effect on the mechanical properties of the 100% wood pulp handsheet. However, the fibers of the NS group were flatter and lost their 3D configuration, resulting in low mechanical strength. Overall, *Rhizoclonium* had its own optimal cooking condition, which was not the same as for wood pulp, and it has potential as a substitute for wood pulp in papermaking.

## 1. Introduction

The pulp and paper industry is an important forest-based global industry, and paper products mostly come from wood. Pulp is a cellulosic fibrous material prepared by chemically or mechanically separating cellulose fibers from wood, fiber crops, waste paper, and other sources, and the pulp is the major material used in paper products [1]. The global pulp and paper market size was valued at 349.18 billion USD in 2020, and the market is expected to grow to USD 370.12 billion by 2028. COVID-19 has disrupted the global supply chains involved in pulp and paper production, including increases in the demand for paper packaging and wrapping paper [2]. With the increases in global demand, the demand for raw materials is also growing. The pulp and paper industry faces growth challenges because of the lack of raw materials. Less than 10% of the raw materials are from recycled fibers [2]. The paper industry consumes a large amount of wood from forests, and the removal of lignin is a major problem in using wood as a raw material [3]. From 2000 to 2015, there was a net loss of 3.3 million hectares of forest area according to the United Nations Food and Agriculture Organization [4]. Therefore, to expand the paper industry, it is necessary to identify other sources of natural fibers.

Algae have very little or no lignin [5]. Past algae use has primarily focused on water-soluble polysaccharide extracts, such as ulvan, agar, carrageenan, alginate, and fucoidan [6]. The residue remaining after extraction is not used effectively, although it contains a large amount of cellulose. Therefore, algae are considered potential natural fiber sources. The cellulose contents of some algae have been determined. Green algae contain 1.5–21.6% cellulose based on dry weight, whereas red algae contain 0.85–34% and brown algae contain 2.2–10.2% cellulose. The contents of algal cellulose vary by species and vary within species [7]. Of these, the variation in cellulose contents for green algae is wider and higher. *Rhizoclonium* is a filamentous green alga that is mainly distributed in subtropical regions and common in culture ponds in Taiwan. It negatively affects the aquaculture industry as it absorbs dissolved oxygen from the water, causing oxygen starvation for cultured animals and preventing them from surfacing for air. Traditionally, *Rhizoclonium* has been removed and discarded using significant manpower. *Rhizoclonium* contains highly crystalline native cellulose with a crystallinity index of 86.5% and comprises 38.6% cellulose [8]. The cellulose fiber length range is 0.2–3.3 mm [9], which is longer than for red algae (0.5–1.0 mm) [10] and brown algae (49–228 nm) [11]. In addition, it grows year-round in Taiwan, and the monthly biomass production reaches 945–1540 kg dry weight/hectare [12]. A recent study indicated that *Rhizoclonium* could replace soybean as the feed source for Nile Tilapia, whereby *Rhizoclonium* replaced 52% of the soybean meal, equivalent to 13% of the total feed, exhibiting a lower total feed intake and daily feed intake but higher specific growth rate than the commercial diet [13]. However, the use of *Rhizoclonium* as a feed additive is still far below the production volume. Therefore, if *Rhizoclonium* was used as a fiber supplement, it could increase the reuse of fishery resources, alleviate land-use demands, and reduce the amount of deforestation.

Fibers are usually made into pulp via chemical cooking procedures, which destroy the fibers’ water-soluble carbohydrates and organic acids [14]. *Rhizoclonium* green alga pulp is prepared with a liquid-to-biomass dry weight ratio of 15, using 5, 10, 15, 20, or 25% NaOH solution with heating at 100 °C for 30 to 120 min [9]. The green alga *Cladophora glomerata* is prepared with a liquid-to-biomass dry weight ratio of 10, using 4% NaClO_2_ in sodium acetate buffer (pH 4.8) with incubation at 60 °C for 3 h. The product is mixed with 0.5 M NaOH at 60 °C overnight, then 5% HCl is added and the mixture is heated until boiling [15]. The *Ulva* green alga pulp is prepared with a liquid-to-biomass dry weight ratio of 9, using 6, 8, or 10% NaOH, 0.2% MgSO_4_, and 0.1% anthraquinone solution with heating at 90 °C for 60 min. The product is further treated with 2, 4, or 6% H_2_O_2_ and 0.5% diethylenetriaminepentaacetic acid (DTPA) and incubated at 60 °C for 60 min [16]. The heating conditions for the algal pulp vary among species, whereas the NaOH solution is only used for *Rhizoclonium* [9]. *Rhizoclonium* has a high cellulose content, with long, crystalline fibers. However, it is not effectively used. Therefore, we demonstrated its utility by preparing algal pulp from *Rhizoclonium* to produce paper samples with good mechanical properties using various cooking conditions.

## 2. Materials and Methods

### 2.1. Rhizoclonium

**Figure 1:**
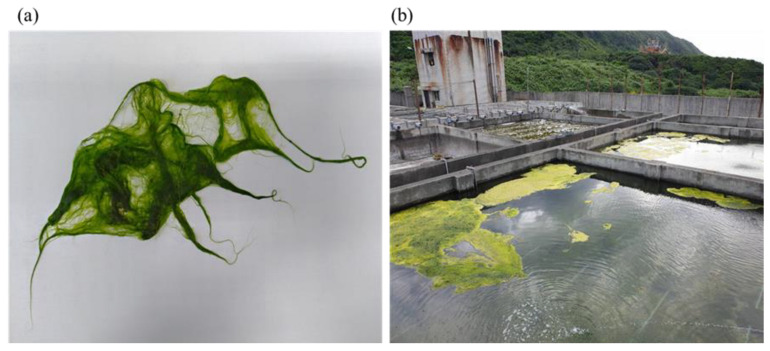
*Rhizoclonium* samples collected from aquaculture ponds: (**a**) green macroalgae *Rhizoclonium*; (**b**) *Rhizoclonium* growth in aquaculture ponds.

The *Rhizoclonium* (Figure 1a) was collected from an aquaculture pond by Dr. Te-Hua Hsu (Department of Aquaculture, National Taiwan Ocean University) at Gongliao District, New Taipei City (Figure 1b). The alga was rinsed with tap water to remove grit and deposits before use, then laid flat and sun-dried until the moisture content was less than 10%.

### 2.2. Rhizoclonium Pulp Cooking Conditions

The clean *Rhizoclonium* (100 g) was cooked with 1.5 L solvent at 95 °C. The solvents included: (1) NaOH (5, 10, or 20%, based on dry algae weight) cooked for 4 h; (2) Na_2_SO_3_ (5, 10, or 20%, based on dry algae weight) cooked for 4 h; (3) NaOH (5, 10, or 20%, based on dry algae weight) cooked for 2 h, and the resulting algal residue was treated with 1% H_2_O_2_ (based on dry algae weight) and heated for 2 h. Table 1 lists the 9 cooking conditions and their parameters. After cooking, the *Rhizoclonium* was separated, washed with distilled water to neutrality, and dried at 50 °C for 8 h as *Rhizoclonium* pulp. Then, 250 g of dry *Rhizoclonium* pulp was added to 8 L of distilled water (approximate 3.57% dry mass basis) in a beater (Vonoya, Taipei, Taiwan) and the blade speed was set to 3000 rpm for 20 min. The length, width, degree of polymerization, and approximate composition were determined. Each parameter was evaluated 3 times.

### 2.3. Composition Analysis

The moisture (method 930.15), crude protein (method 978.04), crude lipid (method 930.09), and ash (method 942.05) contents were analyzed using Association of Official Analytical Chemists procedures [17]. The carbohydrate percentage was determined by subtracting the total percentages of crude protein, crude lipid, and ash on a dry basis from 100. The cellulose content was calculated from the acid detergent fiber (ADF) minus the lignin content. The hemicellulose content was calculated from the neutral detergent fiber (NDF) minus the ADF content. The lignin content was calculated from the ADF minus the ash content [18]. The ADF was analyzed using method #973.18 [19], and the NDF was analyzed using method #992.16 [19]. Briefly, the moisture was determined by drying a weighed amount of the sample in an oven at 135 °C for 2 h and noting the weight loss. The crude protein was determined by using digestion with H_2_SO_4_/Na_2_SO_4_ (1:1) at 420 °C for 2 h, then the digested sample was distilled with NaOH and titrated with 0.1 N HCl. The crude lipid was with ether-extracted in a Soxhlet extractor (Dogger Science, Taipei, Taiwan). The ash was determined after combustion at 600 °C for 4 h in a muffle furnace. The NDF was determined by using digestion with the sample, Na_2_SO_3_, and α-amylase, the residue was washed with hot distilled water and then acetone, then the residue was dried overnight at 100 °C and weighed. The ADF was determined by using digestion with NDF and 72% H2_S_O_4_ for 1 h, the residue was wash with hot distilled water and then acetone, then the residue was dried overnight at 100 °C and weighed.

### 2.4. Characterization of Rhizoclonium Pulp Fiber

Fibers from the *Rhizoclonium* pulp prepared using different cooking conditions were sampled (*n* = 120), and the mean fiber length and width were examined under a light microscope (E400, Nikon, Tokyo, Japan). The degree of polymerization (DP) was determined using standard viscometric methods [20]. The *Rhizoclonium* (100 mg) pulp was dissolved in 100 mL of cupriethylenediamine solvent, and a glass capillary viscosimeter (CT52, Schott and Gen, Mainz, Germany) was used for the analysis. The solution efflux time was measured in duplicate. Martin’s equation was used to calculate the DP value [21].

### 2.5. X-ray Diffraction (XRD) of Rhizoclonium Pulp Fibers

The XRD spectra of the *Rhizoclonium* pulp fibers were analyzed using an XRD meaPanalytical X’Pert Pro MPD (PANalytical, Almelo, The Netherlands). The dried pulp fiber was placed in a diffractometer with a copper target. The diffracted intensity of the radiation was set to 40 kV and 40 mA, and the 2θ° values ranged from 5° to 40°. The crystallinity index % (CI%) was calculated using Segal et al.’s [22] equation: $\mathrm{CI}\%=\frac{I\left(110\right)-\mathrm{Iam}}{I\left(110\right)}\times 100\%$.

Here, I_(110)_ is the intensity of the diffraction (110) lattice at a 2θ° angle close to 22.9°, and Iam is the minimum intensity of (110) lattice at 2θ° angle close to 18.4°.

### 2.6. Handsheet Making

The handsheet was prepared according to CNS method #11212 (CNS Standard). The *Rhizoclonium* pulp prepared using 9 different cooking conditions was formed into handsheets with a 35 ± 2 g/m^2^ basis weight. In substituting wood pulp with *Rhizoclonium* pulp, 0%, 20%, 40%, and 60% 10-NH *Rhizoclonium* pulp samples were mixed with 100%, 80%, 60%, and 40% of commercial wood pulp (Northern Bleached Softwood Kraft long fibered pulp, Chung Rhy Specialty Paper Mfg. Co., Ltd., Puli, Taiwan), designated as 0%, 20%, 40%, and 60% 10-NH mixed pulp group, respectively, and made into handsheets with a 106 ± 2 g/m^2^ basis weight.

### 2.7. Scanning Electron Microscopy (SEM) and Diameter Distribution Analysis

The diameter distribution of the handsheets was analyzed from SEM (Zeiss Sigma, Dresden, Germany) images using ImageJ software 1.8.0. (NIH, Bethesda, MD, USA). The distribution was measured using 80 individual fibers with a 100× magnification.

### 2.8. Fourier Transform Infrared Spectroscopy (FTIR) Analysis

The molecular structure of the handsheets was evaluated using an FTIR-MIDAC 2000 instrument (MIDAC Corporation, Costa Mesa, CA, USA), whereas the FTIR spectrum was recorded at 25 °C in the range of 500–3500 nm.

### 2.9. Atomic Force Microscopy (AFM) and Average Roughness Analysis

The roughness of the handsheets was analyzed via AFM (Bruker, Billerica, MA, USA) using a quartz fiber probe with a conical tip (Nanosensors, Neuchatel, Switzerland) with contact operation. The AFM images were analyzed using NanoScope Analysis v1.40r1 software (Bruker, Billerica, MA, USA). The average roughness was measured under magnification (5 μm × 5 μm).

### 2.10. Mechanical Characterization of the Handsheets

#### 2.10.1. Tensile Index

The tensile index was analyzed according to CNS method #12607 (CNS Standard) using a tensile tester (Thwing-Albert, West Berlin, NJ, USA). The handsheet was cut into 15 mm × 180 mm, and the end of each sample was clamped with paper-based clips. The breaking of the sample occurred within 20 s, and the breaking force value (N) was recorded. The tensile index (N × m/g) = (653.8 × breaking force)/basic weight.

#### 2.10.2. Tear Index

The tear index was analyzed according to CNS method #1355 (CNS Standard) using a tear test (Liansheng Instrument Co., Ltd., New Taipei City, Taiwan). The handsheet was cut to 50 mm × 100 mm. A tear force was applied to a tear length of 45 mm and the tear resistance force (N) was determined. The tear index (N × m/g) = (9.81 × tear resistance force)/basic weight.

#### 2.10.3. Burst Index

The burst index was analyzed according to CNS method #1353 (CNS Standard) using a burst force tester (Liansheng Instrument Co., Ltd., New Taipei City, Taiwan). The handsheet was cut to 10 cm × 10 cm. The burst force was reported in kPa, and the burst index (kPa × m^2^/g) = burst force/basic weight.

#### 2.10.4. Opacity

The opacity of the handsheet was measured according to CNS method #2387 (CNS Standard) with light reflectance of a single sheet backed by a standard backing of reflectance of 0.89.

### 2.11. Statistical Analysis

All data are expressed as the mean ± standard deviation. The results obtained from a one-way analysis of variance and Tukey’s tests were used to analyze the differences among treatments with SPSS software 1.0.0.1406 (IBM, Armonk, NY, USA). The significance level was *p* < 0.05.

## 3. Results and Discussion

### 3.1. Characterization of Rhizoclonium Pulp

The first step in making paper is to use chemicals to extract cellulose from the raw material and convert it into a pulp. Many factors affect the yield and quality of the pulp, including the source, size, and water absorption of the raw material; the composition and concentration of the chemicals; and the operating temperature and time [23]. Reducing the number of chemicals used in the extraction process to obtain a higher yield of cellulose is one of the important goals of pulping. Chemicals that are often used to break the bonds of cellulose fibers are NaOH [24], Na_2_SO_3_ [25], and H_2_O_2_ [26]. Therefore, we determined the effects of different types and amounts of chemicals on the *Rhizoclonium* pulp formation and evaluated the pulping conditions suitable for *Rhizoclonium*. The yields of *Rhizoclonium* pulp obtained with 9 different cooking conditions ranged from 41.1 to 52.5%. Of these, the 5-NS, 10-NS, and 20-NS groups yielded significantly lower (41.1 to 42.5%) amounts and the 5-NH and 10-NH groups resulted in significantly higher (48.3 to 52.5%) amounts. The NH group might have caused less damage to the *Rhizoclonium* after a 2 h exposure to NaOH solvent, so the yield was higher. From the change in pH of the alkaline solvent (NaOH and Na_2_SO_3_) (Table 1), it was evident that the organic acid content in algae is low, enabling alkaline solvents to be used repeatedly to reduce environmental harm [27].

The water content of the cooked algae was lower than that of the raw algae. This was likely the result of cellulose destruction by the solvent and the breaking of hydrogen bonds [28]. *Rhizoclonium* can absorb and remove nitrogen and has been used to treat agro-industrial wastewater [29]. There was approximately 15.9 ± 0.5% crude protein in the raw algae, and the crude protein content of the cooked *Rhizoclonium* was lower than in the raw form. Hedenskog and Hofsten [30] demonstrated that more algal protein could be dissolved in strong alkali, which suggested that the 5-N, 10-N, and 20-N groups contained significantly lower crude protein contents resulting from a long-term strong alkali treatment. In general, macroalgae have a high ash content, including lots of mineral salt and sand, compared with terrestrial plants. The raw *Rhizoclonium* contained 30.7 ± 0.2% ash, which decreased to approximately 20% after cooking (Table 2). Armisen and Galatas reported that the agar extracted from red algae still has about 5% salt, even after complicated purification steps [31]. The patent states that the method for desalting the sea algae is to remove the salt, which has a high ionization strength among contained salts, by turning with direct current electricity in cold water [32]. It was suggested that mineral salts in *Rhizoclonium* were difficult to remove, so the pulp still contained a high amount of mineral salts, while some were dissolved in the solvent. Carbohydrates are a mixture of cellulose, hemicellulose, lignin, and soluble polysaccharides [33]. The sum values of the cellulose and hemicellulose were similar for the 9 different cooking conditions and accounted for 47 to 51% of the total, so the cooking conditions did not appear to affect the total cellulose yield. Trivedi et al. [34] and Baghel et al. [35] reported that the extraction of other algal components before cellulose has no effect on the cellulose yield but may affect the crystallinity of the cellulose [8]. In the present study, none was detected in the lignin of the *Rhizoclonium* (Table 2). After subtracting the cellulose and hemicellulose from the carbohydrate content of the 9 preparations, each group contained approximately 20% water-soluble polysaccharide in the algal pulp. The influence of water-soluble polysaccharides in the process of papermaking remains unclear; however, adding brown algal water-soluble polysaccharides to the wood pulp might increase the mechanical properties of the resulting paper [36].

**Figure 2:**
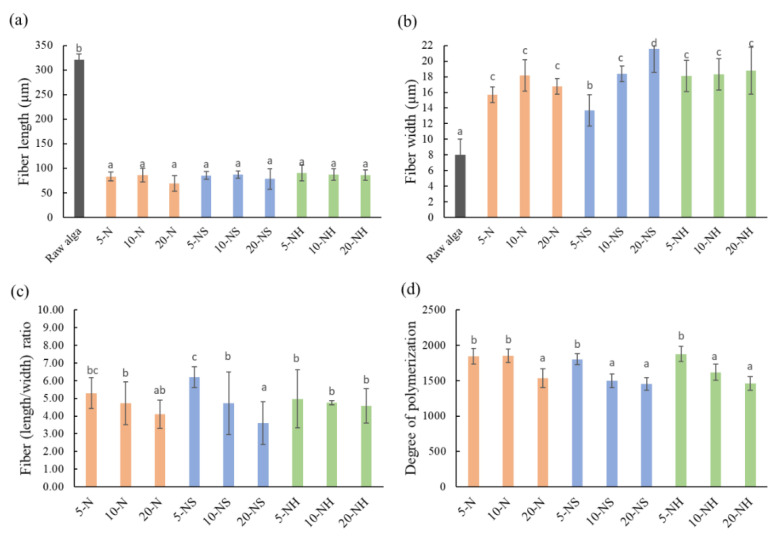
Modification of the *Rhizoclonium* pulp fiber (**a**) length, (**b**) width, (**c**) ratio of length/width, and (**d**) degree of polymerization after cooking and beating treatment. Raw material without cooking and beating treatment. Letters indicate a significant difference within each experiment at *p* < 0.05 in comparison.

A light microscope was used to observe and measure the length and width of the *Rhizoclonium* pulp fiber. The mean fiber length significantly decreased after the cooking treatments (Figure 2a); however, the mean fiber width significantly increased (Figure 2b). In the 5-N, 10-N, and 20-N groups and the 5-NH, 10-NH, and 20-NH groups, the solvent concentration did not significantly affect the fiber width, but in the NS group, the fiber width was positively correlated with the solvent concentration. The fiber length/width ratio is a key microstructural parameter that affects the fiber stiffness and strength. The length is associated with the extensibility, and the width is related to the support force [37]. Before cooking and beating, the *Rhizoclonium* fiber length was 321 ± 12 μm, the width was 8 ± 2 μm, and the length/width ratio was 40.13 ± 1.56. The lengths, widths, and length/width ratios of the *Rhizoclonium* pulp fibers were between 69.4 and 90.2 μm, 13.7 and 21.6 μm, and 3.61 and 6.20, respectively. The length/width ratios of the N and NS groups decreased at higher solvent concentrations. In contrast, the length/width ratio of the NH group did not change significantly because of the solvent concentration (Figure 2c). The wood pulp fiber lengths are between 100 and 300 μm, the widths are between 10 and 50 μm, and the length/width ratio is typically 3 to 5 [38]. Although the length/width ratio of the *Rhizoclonium* pulp fiber and wood pulp fiber was similar, the fiber length and width were lower than that of the wood pulp. This may affect the application of *Rhizoclonium* fiber to pulp and papermaking. The decrease in the DP of the fibers may affect the fiber strength, thereby resulting in decreased tensile and burst strength in the paper. However, a high DP will hinder the fiber dispersion, which will cause difficulty in the papermaking process [39]. We found that the polymerization decreased with higher solvent concentrations of between 1451 and 1878 (Figure 2d), and the *Rhizoclonium* pulp became cotton-like. The decrease in polymerization is primarily the result of the oxidation of hydroxyl-free radicals by the high solvent concentration [40]. From these results, the solvent type and concentration primarily affect the width of the fibers. The fibers in the 20-NS group were the widest, whereas the N groups (5-N, 10-N, and 20-N) exhibited the highest protein removal efficiency.

**Figure 3:**
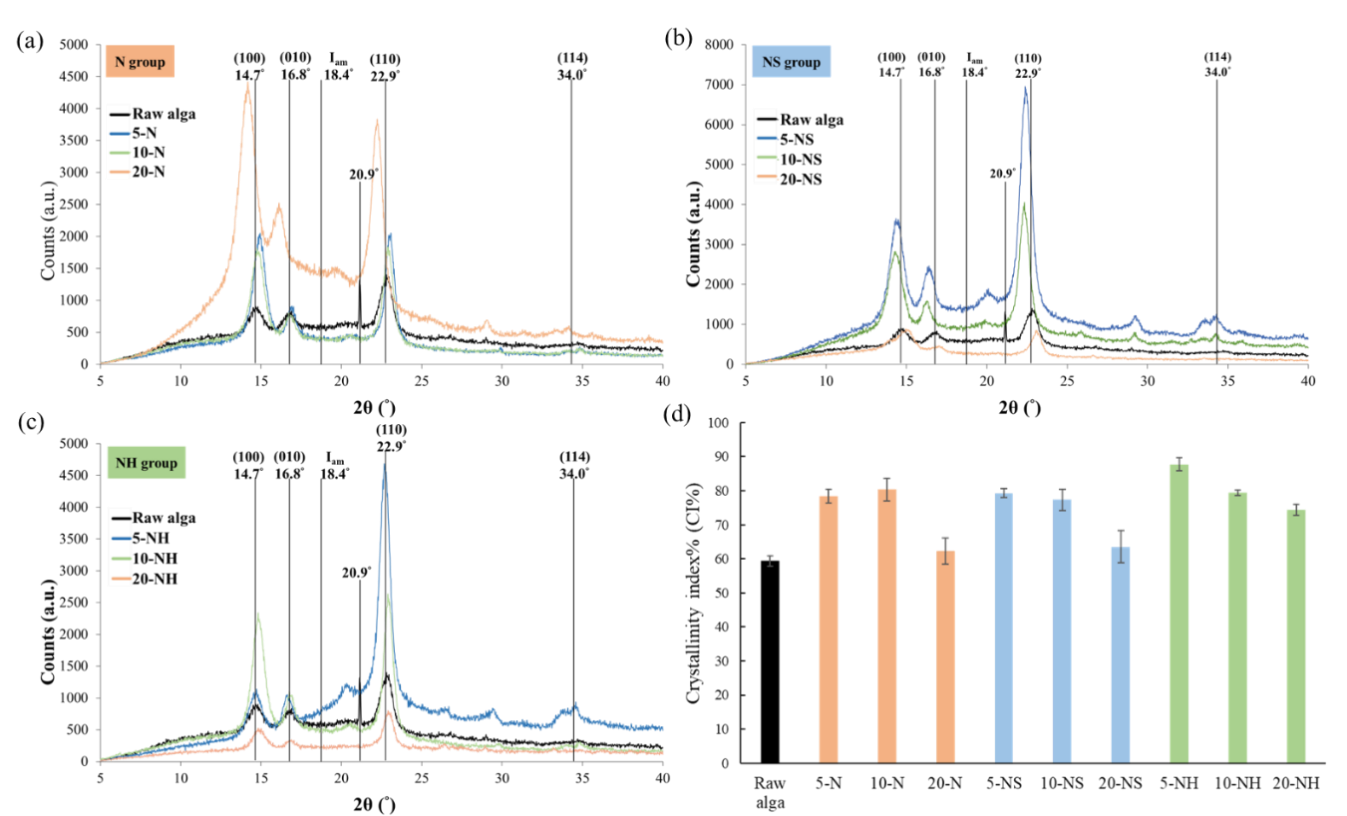
XRD spectra of the (**a**) N group, (**b**) NS group, and (**c**) NH group *Rhizoclonium* pulp samples, and (**d**) the CI% of the *Rhizoclonium* pulp.

The cellulose Iα is the dominant cellulose structure in algae cellulose [41], and the peaks at around 14.5° (100), 16.9° (010), 22.9° (110), and 34.0° (114) correspond to cellulose Iα [42], while the peak at 20.0° corresponds to cellulose II [43]. The main peaks of the raw alga were at 14.7°, 16.8°, 22.9°, and 34.0° corresponding to cellulose Iα, the low amplitude of the peak at 22.9° (110) was at 18.4°, and the CI% was 59.4%. There was a very sharp peak at 20.9° for the raw alga, which we suggest might be caused by the salts in the raw alga, and the peak disappeared after the cooking treatments (Figure 3a–c). The raw alga had a low degree of crystallinity, but after different cooking treatments the CI% increased (Figure 3d), which suggested that the cooking solvent might dissolve the non-cellulose components, while the cellulose long chains rearrange for better crystallinity. The change in CI% after the alkali treatment echoed the findings of Chao, Su and Chen [9]. Using the same alkali solvent concentration, the CI% of the NH group was greater compared with that of the N group and NS group, and the CI% decreased with higher solvent concentrations (Figure 3d). The fibers were more crystalline, have a higher tensile strength and stiffness but lower elongation capabilities [44], which might affect the properties and substitution amount of the algal pulp. The morphological change in cellulose I to cellulose II in sugar beet (higher plant) is significant when the NaOH concentration is increased by more than 9% [45]. However, in this study, there was no similar result for cellulose I conversion into cellulose Π when the alga was cooked in 20% NaOH at 95 °C for 4 h. Shibazaki et al. [46] demonstrated that cotton cellulose (higher plant) is converted to cellulose II with fairly high crystallinity using an alkali treatment for as little as 3 min, but bacterial cellulose keeps its morphology after treatment with NaOH solutions of less than 9%. The change occurred when treated with more than 12% NaOH and with the conversion of crystals to cellulose II, while the crystallinity was increased significantly by elongated treatment up to 10 days. Bhutiya et al. [47] also used the Segal et al. [22] equation to calculate the CI% of cellulose in seaweed and Cu_2_O-nanorod-deposited seaweed, and the crystallinity of cellulose had improved during the chemical extraction process as compared to raw seaweed. The intensity of the seaweed cellulose is affected by the Cu_2_O deposition [47]. We, therefore, suggest that although the 20.9° peak generated by the ash component is not within the formula parameters, it might still affect the value of I (110) or Iam.

### 3.2. Characterization of Handsheets Produced from the Algal Pulp

**Figure 4:**
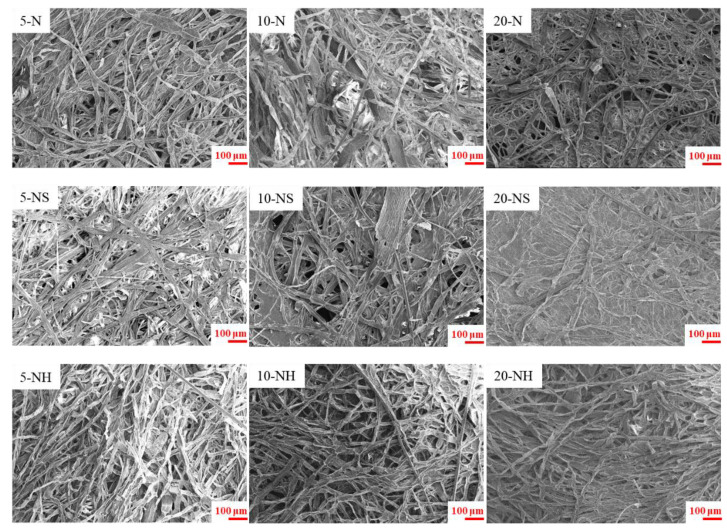
SEM images of *Rhizoclonium* pulp under different cooking treatments in handsheets.

**Figure 5:**
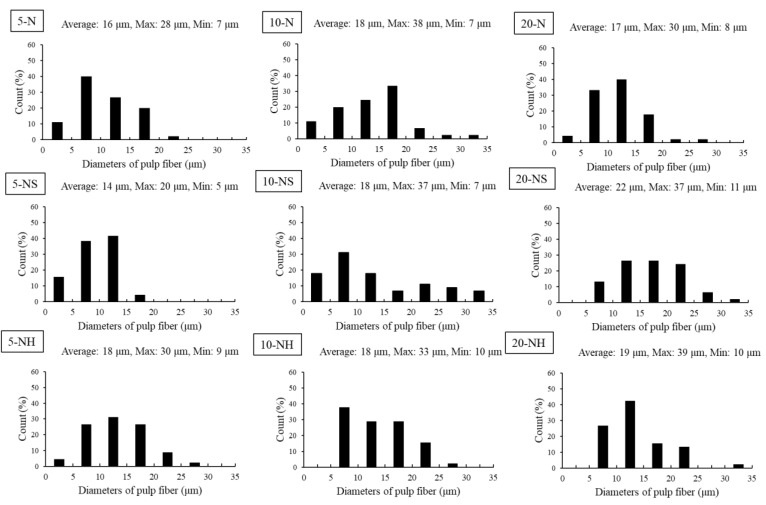
Diameter distribution of *Rhizoclonium* pulp fibers under different cooking treatments in handsheets. The distribution was measured using 80 individual fibers at 100× magnification.

**Figure 6:**
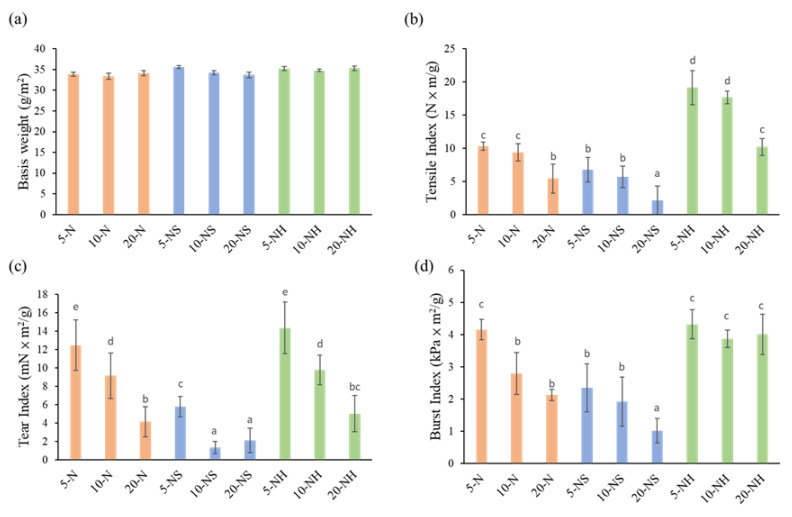
(**a**) Basic weights, (**b**) tensile index, (**c**) tear index and (**d**) burst index of *Rhizoclonium* pulp samples under different cooking treatments in handsheets. Letters indicate a significant difference within each experiment at *p* < 0.05 in comparison.

The above 9 types of pulp were made into handsheets to analyze the paper’s morphology, chemical, and physical properties. The morphology of a handsheet is shown in the SEM images (Figure 4). The higher solvent concentration resulted in flatter fibers on the surface of the handsheet, and the three-dimensional characteristics of the fibers were less obvious. Among the N, NS, and NH groups, the fibers of the NS group were the flattest, particularly in the 20-NS group, in which the fibers had almost lost their three-dimensional configuration and the web-like structure associated with previous studies of the *Rhizoclonium* cellulose structure [9]. The diameters of 5-N, 10-N, and 20-N were less than 20 μm; the diameter of 5-NS was less than 15 μm; and the diameters of 5-NH, 10-NH, and 20-NH were between 5 and 20 μm. However, the diameters of 10-NS and 20-NS were wider and ranged from 0 to 35 μm (Figure 5). The basis weights of the 9 handsheet types were between 33.4 and 35.6 g/m^2^. The mechanical property analysis showed that the tensile, tear, and burst index values decreased with higher alkali solvent concentrations. Using the same alkali solvent concentration, the tensile, tear, and burst index values of the NH group were greater compared with the N group. In contrast, the NS group had the worst mechanical properties (Figure 6). This may have been associated with the large difference and wide distribution of the fiber diameters in the NS group (Figure 5), which resulted in an uneven support force of the web-like structure and low mechanical strength [48]. In addition, the lower crystallinity of the NS group might also result in low mechanical strength (Figure 3).

Despite their better mechanical specificity, the fibers of the 5-N, 5-NS, and 5-NH groups did not readily separate fibers during handsheet production. Chao, Su and Chen [9] applied a single chemical reagent when cooking green alga pulp using 5, 10, 15, 20, or 25% NaOH solutions. Xiang, Gao, Chen, Lan, Zhu and Runge [15] reported 0.5 M NaOH with 4% NaClO_2_ as a major chemical reagent for cooking green alga pulp. Moral, Aguado, Castelló, Tijero and Ballesteros [16] recommended 6, 8, or 10% NaOH with 2, 4, or 6% H_2_O_2_ and a small number of other chemicals as the cooking solvents for green alga pulp. Lakshmi et al. [49] used 2% NaOH as a cooking solvent for red alga pulp, whereas only Seo et al. [50] used 0.5% H_2_SO_4_ to cook red alga pulp. The cooking solvent for algal pulp requires primarily alkaline reagents.

Although Na_2_SO_3_ is a commonly used cooking solvent for terrestrial plants [51], no studies discuss the feasibility of Na_2_SO_3_ as a cooking solvent for algae. The results of our study, however, indicate that the Na_2_SO_3_ pretreatment widens the fiber diameter (Figure 2b), has a wide distribution (Figure 5), and eliminates the three-dimensional configuration of the handsheets (Figure 4). Therefore, we suggested that Na_2_SO_3_ is not an appropriate cooking solvent for *Rhizoclonium*. The obvious difference between the N group and NH group was in the cooking times (4 h vs. 2 h, respectively). The tensile index indicates the elongation of the paper, which means how much it can stretch before tearing. The tear index represents the force needed to rip a material and to make the crack continue until it fails. The burst index represents the capacity of the paper to maintain under continuity force. Since the above three parameters will be affected by the basis weight of the paper, all handsheets were analyzed under similar basis weights (Figure 6a). The tension, tear, and burst index values of the N group and NS group were significantly lower than those of the NH group, and the tension, tear, and burst index values decreased with higher solvent concentrations (Figure 6b–d). Long-term and highly concentrated alkali treatment breaks the fiber structure and reduces its strength [24]; thus, it was suggested that the 5-NH and 10-NH groups are more suitable as cooking treatments for *Rhizoclonium* pulp. However, during practical operation, the 10-NH pulp was easier to separate compared with the 5-NH pulp, so the 10-NH pulp was selected as the follow-up analysis.

**Figure 7:**
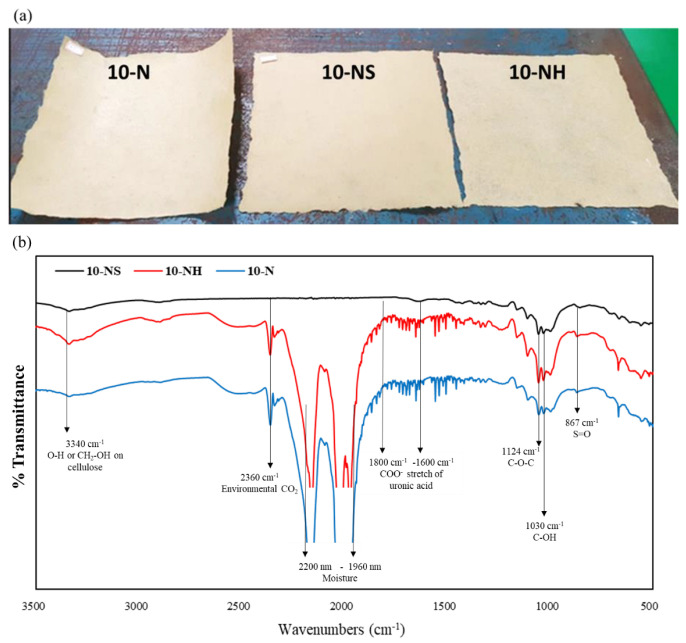
(**a**) Visual appearance and (**b**) FTIR spectra of *Rhizoclonium* pulp samples prepared using 10-N, 10-NH, and 10-NS cooking treatments in handsheets.

FTIR can provide important information about the functional groups present in compounds and complex substances. Its vertical axis is in % transmittance, and a transmittance percentage of 100 means that all frequencies pass directly through the compound without being absorbed. The 10-N, 10-NH, and 10-NS handsheets (Figure 7a) exhibited clear S=O absorption peaks at 867 cm^−1^ for green alga polysaccharides [52]. The peaks at 1124 cm^−1^ and 1030 cm^−1^ resulted from the C-O-C and C-OH of the polysaccharide [53], whereas the peaks at 3340 cm^−1^ were from O-H or CH_2_-OH groups on cellulose [53]. These absorption peaks indicate that the three *Rhizoclonium* pulps are not pure cellulose but contain some water-soluble polysaccharides. Peaks in the range of 1600–1800 cm^−1^ indicate a COO^-^ stretch of uronic acid in green alga polysaccharides [52], but they only appeared in the 10-N and 10-NH handsheets (Figure 7b). The NaOH treatment may soften the *Rhizoclonium* cell wall but not destroy it, so uronic acid can still be detected in the 10-N and 10-NH handsheets. The peaks at 1960–2200 cm^−1^ and 2360 cm^−1^ were probably caused by water uptake and environmental CO_2_ molecules [54], which only occurred in the 10-N and 10-NH handsheets. Chao et al. [9] prepared *Rhizoclonium* pulp using a NaOH solvent that had similar peaks. Forney and Brandl [55] concluded that humidity is an important environmental factor that affects the physical properties of the whole plant and its derived products. For example, the plant fibers are hard and brittle in a relatively dry environment and more elastic in a relatively humid environment. Therefore, the handsheets prepared from NaOH-treated pulp may be more susceptible to moisture; however, their hygroscopic properties may also improve their mechanical properties. As shown in Figure 6, the mechanical properties of the N and NH group handsheets were better compared with that of the NS group. We suggest that the 10-NH conditions were the best among the 9 cooking groups based on the above results.

### 3.3. Characterization of Handsheets Produced from Algal Pulp Mixed with Wood Pulp

**Figure 8:**
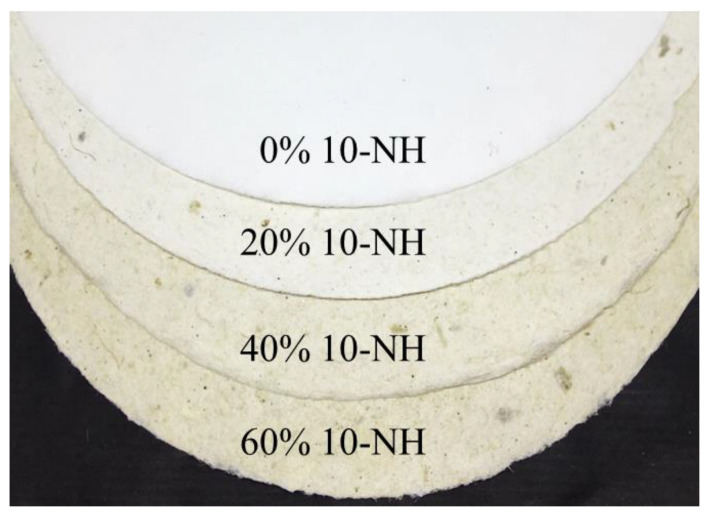
Visual appearance of 0%, 20%, 40%, and 60% 10-NH mixed pulp handsheets.

The handsheets made from *Rhizoclonium* pulp generally exhibit weak mechanical properties compared with wood pulp. The wall structure of the wood pulp is primarily composed of microfibrils that improve the bonding and strength between the fibers. However, algal pulp lacks microfibrils, which results in the paper having low mechanical strength [9,56]. Chao, Su and Chen [9] improved the mechanical properties of a handsheet made from *Rhizoclonium* pulp by mixing it with wood pulp. The results showed that the strength properties of the handsheet were improved by combining 10% *Rhizoclonium* pulp (20% NaOH cooking solvent) with 90% wood pulp. In order to reduce the consumption of wood pulp resources and to use *Rhizoclonium* pulp effectively, the wood pulp was mixed with 0%, 20%, 40%, and 60% 10-NH *Rhizoclonium* pulp to prepare handsheets that were analyzed for their mechanical properties, morphology, and surface roughness. The basis weights of the 5 types of handsheet were between 35.8 and 37.8 g/m^2^ (Figure 8). A higher proportion of 10-NH *Rhizoclonium* pulp resulted in lower mechanical properties in the handmade paper and higher opacity. When the amount of 10-NH *Rhizoclonium* pulp increased to 40%, the mechanical properties of the handmade paper decreased significantly. In contrast, adding 20% 10-NH *Rhizoclonium* pulp had no significant effect on the mechanical properties of the handmade paper (Table 3). The opacity is expressed as a percentage and is a measure of the light transmittance of the paper. It also represents the ability of the paper to hide or mask colors or objects on the back of the paper. The high opacity of the paper allows the front of the page to be viewed without being distracted by the printed image on the back. Therefore, opacity was used in this experiment to analyze the mixed pulp in the handsheets. From the opacity (%) value in Table 3, it can be seen that the more *Rhizoclonium* pulp is added, the higher the opacity (%) value and the higher the opaqueness. It is suggested that adding *Rhizoclonium* pulp helped reduce the front of the page from distracting from the printed image on the back.

**Figure 9:**
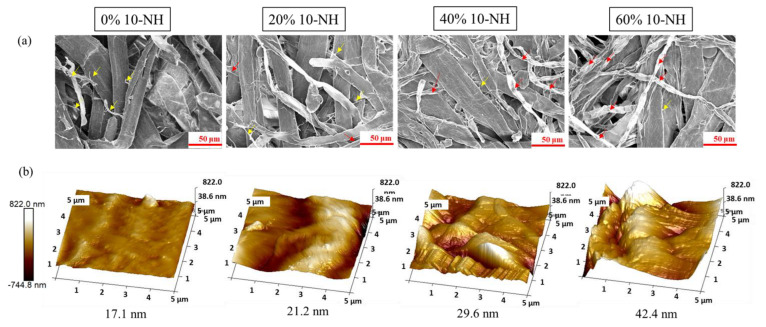
(**a**) SEM and (**b**) AFM images with average roughness values for 0%, 20%, 40%, and 60% 10-NH mixed pulp handsheets (red arrow: 10-NH algal fiber; yellow arrow: microfibrils of wood fiber).

Based on the SEM images, the 10-NH *Rhizoclonium* fibers (Figure 9a, red arrow) and wood pulp fibers were interleaved, and the wood pulp fibers were thicker than the 10-NH *Rhizoclonium* fibers. There were microfibrils (Figure 9a, yellow arrow) around the wood pulp fibers, but not the 10-NH *Rhizoclonium* fibers (Figure 9a). The X and Y axes of the AFM images represent the sample analysis area (5 μm × 5 μm). The Z axis represents the height of the sample, and the height was selected within the range of −744.8–822.0 nm. The values were plotted in a pseudocolor image, with brighter colors representing higher heights, and vice versa. The AFM three-dimensional images of the rough and smooth faces of the 10-NH mixed pulp in handsheets is shown in Figure 9b. The higher the amount of 10-NH *Rhizoclonium* pulp, the higher the roughness of the handmade paper, whether rough or smooth. Kılıç et al. [57] demonstrated that the surface roughness decreases and the tensile strength of the polyvinyl chloride membrane increases, but the increase is not linear. Thus, when the amount of 10-NH *Rhizoclonium* pulp increased to 40%, the surface roughness of the handsheet increased, the height difference of the surface was larger, and the load-carrying capability was not uniform, which reduced the mechanical properties of the handsheet.

## 4. Conclusions

*Rhizoclonium* has long fibers, high crystalline and cellulose contents, and high yield characteristics. We examined the potential use and value of *Rhizoclonium* as a raw material for papermaking. Since papermaking is an industry with a long history, it is a big challenge to change the operation process, solvent, equipment, and other factors. Therefore, three kinds of chemicals commonly used in wood fiber, namely NaOH, Na_2_SO_3_, and H_2_O_2_, were selected for the experiment. Although Na_2_SO_3_ mainly plays a role in delignification, it still breaks the bonds of the cellulose to soften the fibers. The present study had the aim of accelerating the application of *Rhizoclonium* in the current paper industry with minimal factor changes.

The total cellulose and hemicellulose contents of the *Rhizoclonium* pulp samples were similar in the 9 different groups and were not dependent on the cooking conditions (Table 2). After cooking, the mean fiber length significantly decreased and the mean fiber width significantly increased, whereas the fibers of the NS handsheet group were flatter and they lost their three-dimensional configuration and web-like structure, resulting in lower mechanical strength (Figure 2, Figure 4, and Figure 6). However, the CI% was increased after the cooking treatment, indicating that the pulp cooking treatment helped to improve the crystallinity and possibly some of the mechanical properties (Figure 3). The difference between the N and NH groups was that the alkali treatment time of the N group was longer. The mechanical properties of the NH group handsheet were superior to that of the N group (Figure 6), indicating that long-term alkali treatment may break the fiber structure and reduce the handsheet strength. Although the 5-NH handsheet showed the best mechanical properties, the 10-NH pulp was easier to separate than 5-NH during handsheet making, and 10-NH was more suitable for the industrial process. It was suggested that the 10-NH condition was the best among the 9 cooking conditions tested. Overall, *Rhizoclonium* had its own optimal cooking condition, which was not the same as wood pulp. In the substitution experiment, substitution with 20% 10-NH *Rhizoclonium* pulp had no significant effect on the mechanical properties of the handsheet, so 10-NH *Rhizoclonium* pulp can effectively replace 20% of the wood pulp to reduce the consumption of wood pulp resources.

## Figures and Tables

**Table 1:** Cooking conditions and solid yield of *Rhizoclonium*.

Term	Chemicals *	Temperature (°C)	Cooking Time (h)	pH Level of Solvent from Initial to End	Yield (%)
5-N	5% NaOH	95	4	13.5–12.2	49.4 ± 0.3 ^b^
10-N	10% NaOH	95	4	13.8–12.7	48.9 ± 0.4 ^b^
20-N	20% NaOH	95	4	13.9–13.1	47.8 ± 0.1 ^b^
5-NS	5% Na_2_SO_3_	95	4	9.6–9.7	42.5 ± 0.5 ^c^
10-NS	10% Na_2_SO_3_	95	4	10.1–9.5	42.4 ± 0.2 ^c^
20-NS	20% Na_2_SO_3_	95	4	11.3–9.6	41.1 ± 0.5 ^c^
5-NH	5% NaOH	95	2	13.5–11.8	52.5 ± 0.2 ^a^
	1% H_2_O_2_	95	2	4.2–8.6	
10-NH	10% NaOH	95	2	13.8–12.6	52.1 ± 0.3 ^a^
	1% H_2_O_2_	95	2	4.2–8.3	
20-NH	20% NaOH	95	2	13.9–12.8	48.3 ± 0.2 ^b^
	1% H_2_O_2_	95	2	4.2–9.0	

* Based on dry algae weight. Letters indicate a significant difference within each experiment at *p* < 0.05 in comparison.

**Table 2:** Moisture and proximate composition (% dry weight) of raw *Rhizoclonium* and *Rhizoclonium* pulp samples after cooking.

	Moisture	Crude Protein	Crude Lipid	Ash	Carbohydrate	Cellulose	Hemicellulose	Lignin
Raw alga	12.8 ± 0.1 ^a^	15.9 ± 0.5 ^a^	0.8 ± 0.3	30.7 ± 0.2 ^a^	52.6 ± 0.6 ^a^	18.4 ± 0.1 ^a^	5.6 ± 0.2 ^a^	-
5-N	10.2 ± 0.4 ^b^	8.3 ± 0.2 ^c^	0.8 ± 0.1	21.4 ± 0.3 ^b^	69.5 ± 0.2 ^b^	35.8 ± 0.1 ^b^	12.5 ± 0.4 ^c^	-
10-N	9.9 ± 0.2 ^b^	7.9 ± 0.3 ^c^	0.7 ± 0.3	21.6 ± 0.5 ^b^	69.8 ± 0.2 ^b^	36.4 ± 0.5 ^b^	12.1 ± 0.5 ^c^	-
20-N	10.5 ± 0.1 ^b^	8.2 ± 0.4 ^c^	0.8 ± 0.2	20.5 ± 0.2 ^b^	70.5 ± 0.5 ^b^	36.1 ± 0.3 ^b^	10.8 ± 0.2 ^b^	-
5-NS	10.2 ± 0.1 ^b^	10.5 ± 0.2 ^b^	0.8 ± 0.3	21.5 ± 0.3 ^b^	67.2 ± 0.2 ^c^	35.5 ± 0.3 ^b^	10.5 ± 0.3 ^b^	-
10-NS	9.8 ± 0.3 ^b^	11.9 ± 0.3 ^b^	0.8 ± 0.1	20.3 ± 0.1 ^b^	67.0 ± 0.3 ^c^	36.1 ± 0.2 ^b^	11.2 ± 0.5 ^bc^	-
20-NS	10.3 ± 0.2 ^b^	11.4 ± 0.4 ^b^	0.8 ± 0.2	20.4 ± 0.2 ^b^	66.2 ± 0.5 ^c^	36.2 ± 0.3 ^b^	11.8 ± 0.2 ^c^	-
5-NH	10.2 ± 0.3 ^b^	10.1 ± 0.4 ^b^	0.8 ± 0.2	21.9 ± 0.4 ^b^	67.2 ± 0.2 ^c^	35.9 ± 0.8 ^b^	11.9 ± 0.4 ^bc^	-
10-NH	10.1 ± 0.3 ^b^	10.3 ± 0.2 ^b^	0.8 ± 0.2	21.8 ± 0.5 ^b^	67.1 ± 0.3 ^c^	36.4 ± 0.4 ^b^	10.5 ± 0.3 ^b^	-
20-NH	10.4 ± 0.2 ^b^	10.9 ± 0.3 ^b^	0.8 ± 0.1	20.7 ± 0.1 ^b^	67.6 ± 0.5 ^c^	35.4 ± 0.2 ^b^	12.6 ± 0.5 ^c^	-

Letters indicate a significant difference within each experiment at *p* < 0.05 in comparison.

**Table 3:** Mechanical properties of 0%, 20%, 40%, and 60% 10-NH mixed pulp handsheets.

	Basis Weight (g/m^2^)	Tensile Index (N × m/g)	Tear Index (mN × m^2^/g)	Burst Index (kPa × m^2^/g)	Opacity (%)
0% 10-NH	35.8 ± 1.2	38.24 ± 2.31 ^a^	17.86 ± 0.91 ^a^	15.16 ± 0.65 ^a^	94.0 ± 0.8
20% 10-NH	36.4 ± 0.9	37.50 ± 1.65 ^a^	17.46 ± 1.25 ^a^	14.06 ± 0.55 ^a^	96.5 ± 0.6
40% 10-NH	36.6 ± 1.4	28.74 ± 1.84 ^b^	12.76 ± 2.57 ^b^	10.85 ± 0.41 ^b^	98.4 ± 0.7
60% 10-NH	37.8 ± 1.5	17.88 ± 0.96 ^c^	5.79 ± 2.14 ^c^	4.71 ± 0.39 ^c^	99.1 ± 1.5

Letters indicate a significant difference within each experiment at *p* < 0.05 in comparison.

## Data Availability

Not applicable.
